# Therapeutic cancer vaccination against mutant calreticulin in myeloproliferative neoplasms induces expansion of specific T cells in the periphery but specific T cells fail to enrich in the bone marrow

**DOI:** 10.3389/fimmu.2023.1240678

**Published:** 2023-08-17

**Authors:** Morten Orebo Holmström, Morten Andersen, Sofie Traynor, Shamaila Munir Ahmad, Thomas Landkildehus Lisle, Jacob Handlos Grauslund, Vibe Skov, Lasse Kjær, Johnny T. Ottesen, Morten Frier Gjerstorff, Hans Carl Hasselbalch, Mads Hald Andersen

**Affiliations:** ^1^ National Center for Cancer Immune Therapy, Department of Oncology, Herlev University Hospital, Herlev, Denmark; ^2^ Department of Immunology and Microbiology, University of Copenhagen, Copenhagen, Denmark; ^3^ Centre for Mathematical Modeling – Human Health and Disease, IMFUFA, Department of Science and Environment, Roskilde University, Roskilde, Denmark; ^4^ Department of Cancer and Inflammation Research, Institute of Molecular Medicine, University of Southern Denmark, Odense, Denmark; ^5^ Department of Hematology, Zealand University Hospital, Roskilde, Denmark; ^6^ Department of Oncology, Odense University Hospital, Odense, Denmark

**Keywords:** myeloproliferative neoplasms, calreticulin, cancer vaccines, immune escape, adaptive immunity

## Abstract

**Background:**

Therapeutic cancer vaccination against mutant calreticulin (*CALR*) in patients with *CALR*-mutant (*CALR*mut) myeloproliferative neoplasms (MPN) induces strong T-cell responses against mutant CALR yet fails to demonstrate clinical activity. Infiltration of tumor specific T cells into the tumor microenvironment is needed to attain a clinical response to therapeutic cancer vaccination.

**Aim:**

Determine if CALRmut specific T cells isolated from vaccinated patients enrich in the bone marrow upon completion of vaccination and explore possible explanations for the lack of enrichment.

**Methods:**

CALRmut specific T cells from four of ten vaccinated patients were expanded, enriched, and analyzed by T-cell receptor sequencing (TCRSeq). The TCRs identified were used as fingerprints of CALRmut specific T cells. Bone marrow aspirations from the four patients were acquired at baseline and at the end of trial. T cells were enriched from the bone marrow aspirations and analyzed by TCRSeq to identify the presence and fraction of CALRmut specific T cells at the two different time points. *In silico* calculations were performed to calculate the ratio between transformed cells and effector cells in patients with *CALR*mut MPN.

**Results:**

The fraction of CALRmut specific T cells in the bone marrow did not increase upon completion of the vaccination trial. In general, the T cell repertoire in the bone marrow remains relatively constant through the vaccination trial. The enriched and expanded CALRmut specific T cells recognize peripheral blood autologous *CALR*mut cells. *In silico* analyses demonstrate a high imbalance in the fraction of *CALR*mut cells and CALRmut specific effector T-cells in peripheral blood.

**Conclusion:**

CALRmut specific T cells do not enrich in the bone marrow after therapeutic cancer peptide vaccination against mutant CALR. The specific T cells recognize autologous peripheral blood derived *CALR*mut cells. *In silico* analyses demonstrate a high imbalance between the number of transformed cells and CALRmut specific effector T-cells in the periphery. We suggest that the high burden of transformed cells in the periphery compared to the number of effector cells could impact the ability of specific T cells to enrich in the bone marrow.

## Introduction

The chronic myeloproliferative neoplasms (MPN) are neoplastic diseases of the hematopoietic stem cells in the bone marrow (1). The three types of MPN are essential thrombocythemia (ET), polycythemia vera and primary myelofibrosis (PMF). The mutational landscape in MPN is highly heterogenous as approximately 55% of patients harbor the Janus kinase 2 (*JAK2*)V617F mutation (2), and 20% of patients a mutation in exon 9 of the calreticulin (*CALR*) gene (3, 4). Even though more than 50 types of *CALR* mutations have been identified, they all generate a 36 amino acid sequence in the C-terminus of the CALR protein that is shared between all patients (5). We have demonstrated that the mutant C-terminus of mutant CALR is a cancer neo-antigen as the mutations are immunogenic (6), and both CD4^+^ and CD8^+^ T cells specific for peptides derived from the mutant CALR C-terminus recognize and kill autologous *CALR*mut cells (7, 8). Given the high immunogenicity of the mutations, and the identification of CALRmut specific T-cell memory responses in healthy donors (9), which suggest a defect in the tumor immune surveillance in *CALR*mut patients (10), we conducted a phase I clinical vaccination trial against mutant CALR (NCT 03566446) (11). In this trial, 10 patients with *CALR*mut MPN received 15 vaccines over the course of one year. The vaccines consisted of a peptide (CALRLong36) derived from the mutant CALR C-terminus with montanide as an adjuvant. The vaccines were safe and tolerable and induced T-cell responses to the vaccination epitope. Curiously, none of the patients displayed neither a hematological nor a molecular response to the vaccines (11). The failure of the vaccines may be attributed to several mechanisms that were recently reviewed (12). Yet, the lack of response could be ascribed to failure of CALRmut specific T cells to enrich in the bone marrow. In the present study we used T-cell receptor sequencing (TCRSeq) to investigate the homing of CALRmut specific T cells to the bone marrow in four vaccinated patients and show that the specific T cells do not home to the bone marrow. CALRmut specific T cells recognize autologous *ex vivo* isolated peripheral blood (PB) *CALR*mut monocytes which made us hypothesize that the tumor burden in PB outnumbers the specific T cells. This hypothesis was confirmed by in *silico* analyses as we showed a high imbalance between the number of transformed cells in *CALR*mut MPN compared to the amount of effector immune cells in patients, thus demonstrating that the burden of transformed cells in MPN is an important obstacle to overcome for cancer immune therapy to have effect in patients with MPN.

## Methods

### Establishment of specific T-cell cultures and sorting of T cells

**Figure 1:**
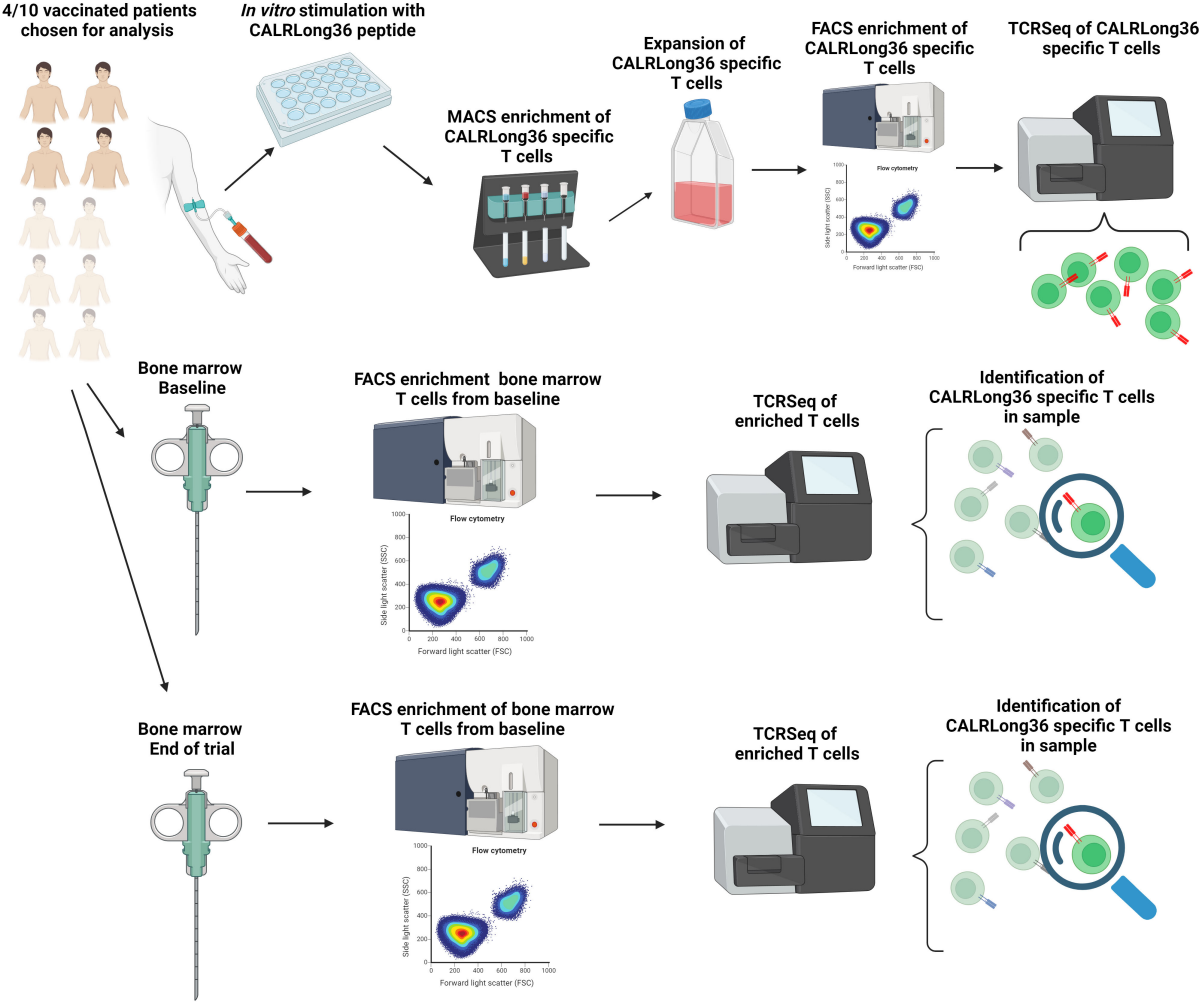
Visual abstract of the methods employed for the T cell receptor sequencing in this study. Four patients with a strong vaccine induced immune response to the CALRLong36 epitope were chosen as donors for expansion of CALRmut specific T cells. Firstly, patient peripheral blood mononuclear cells were stimulated *in vitro* with CALRLong36 peptide. Next CALRLong36 specific T cells were enriched using magnetically activated cells sorting of cytokine secreting cells, and the enriched cells were expanded using our rapid expansion protocol. Expanded cells were then restimulated with CALRLong36 peptide and enriched using fluorescense activated cells sorting (FACS) by gating on CD137^+^/CD107a^+^ double positive cells. Enriched cells were subjected to T-cell receptor sequencing (TCRSeq) which allowed us to identify sequence of the TCR in CALRmut specific T cells (*Top, left to right*). Bone marrow aspirations from the four patients were isolated at trial baseline and at end of trial. Bone marrow derived T cells from the aspirations were enriched using FACS by gating on live CD3^+^ events. The enriched cells were analyzed by TCRSeq and through our analyses of the TCR of CALRmut specific T cells above we ascertained the presence and fraction of CALRmut specific T cells (red TCR) in the bone marrow at baseline and at the end of the trial (*Middle and below, left to right*). Created with BioRender.com.

Four patients (three patients with ET and one patient with post-ET myelofibrosis) that completed our CALRmut vaccination trial and displayed an immune response to the CALRLong36 epitope were chosen as donors for generation of specific T-cell cultures. Cultures were generated as previously described by stimulation with the CALRLong36 peptide (13). Specific T cells were enriched by overnight incubation after restimulation with CALRLong36 peptide and labelled with fluorescence labelled antibodies after 18 hours of stimulation. Specific T cells were sorted using a fluorescence activated cell sorting (FACS) Melody cell sorter (BD Biosciences, San José, CA, USA) with the sorting gate set on live, CD3^+^, CD4^+^ or CD8^+^, CD137^+^/CD107a^+^ double positive events (**Supplementary Material 1**, top). Enrichment purity was assessed, and pellets of specific cells were kept at -80 °C. Sorting of bone marrow derived T cells was performed on cryopreserved bone marrow derived mononuclear cells. T cells were enriched as live CD3^+^ positive events (**Supplementary Material 2**, bottom). Enrichment purity was assessed after FACS, and enriched T cells were pelleted and cryopreserved as described above. A visual abstract of the methods described above is provided in **Figure 1**. A list of fluorescence labelled antibodies is provided in **Supplementary Material 2**.

### FACS sorting and ELISPOT analyses of T-cell reactivity to autologous peripheral blood cells

Autologous peripheral blood lymphocytes (PBL) and monocytes were enriched using a BD FACS Melody live cell sorter with monocytes sorted as live CD14^+^ events, and PBL sorted as live CD14^-^ events (**Supplementary Material 3**). The sorted cells were setup in an IFN-γ Enzyme Linked ImmunoSPOT (ELISPOT) assay which was coated, developed and analyzed as previously reported (13). In short, CALRmut specific T cells were aliquoted in 10^5^ aliquots in triplicates. T cells were stimulated with either CALRLong36 peptide (positive control), 10^4^ autologous monocytes, 10^4^ autologous PBL, or left unstimulated (negative control). To assess the cytokine activity from monocytes and PBL, 10^4^ autologous monocytes and 10^4^ autologous PBL were plated alone in triplicate wells serving as an additional negative control. Normalized counts were calculated by subtracting the spot count in the wells with unstimulated T cells and the wells with monocytes and PBL respectively from the spot count in wells with monocyte stimulated T cells and PBL stimulated T cells respectively.

### Flow cytometric analysis of HLA-I and HLA-II expression by monocytes and PBL

Patient peripheral blood mononuclear cells (PBMC) were thawed and treated with Fc-block (Human TruStain FcX receptor blocking solution, Biolegend, San Diego, CA, USA) and stained with fluorescent antibodies (**Supplementary Material 2**). Cells were acquired on a Novocyte Quanteon Flow cytometer (Agilent, Santa Clara, CA, USA) for the assessment of HLA-I expression, and a FACS Canto II flow cytometer (BD Biosciences, San José, CA, USA) for assessment of HLA-II, both with appropriate compensation controls. Mean fluorescence intensity (MFI) for HLA-I and HLA-II was calculated by subtracting the MFI of the isotype control sample from the HLA-I/II stained sample.

### *CALR*mut analysis by digital droplet PCR

Performed as previously described (14, 15).

### T-cell receptor sequencing

Total RNA purification was performed on cell pellets of CALRLong36-specific T cells or bone marrow derived T cells using the RNEasy Plus Mini Kit (Qiagen, Germantown, MD, USA) according to the manufacturer’s supplied protocol. RNA concentration was measured on a NanoDrop2000 Spectrophotometer (Thermo Scientific, Waltham, MA, USA). T cell receptor sequencing was performed using the SMARTer Human TCR a/b Profiling kit (Takara 635016, San Jose, CA, USA) according to the manufacturer’s protocols. The TCR libraries were sequenced on an Illumina NovaSeq 6000 platform (Illumina, San Diego, CA, USA) using read lengths of 150 bp read 1, 8 bp i7 index, 150 bp read 2, 8 bp i5 index and 20% PhiX. TCR reads were analyzed using the Cogent NGS Immune Profiler Software v1.0 which enable data preprocessing, UMI based analysis, clonotype calling and statistical analysis. Output from the Cogent Profiler was further processed in R using the software package Immunarch (16). Clonotypes with less than two reads were excluded from further analysis. To filter out redundant TCR clonotypes identified in the specific T-cell cultures, clonotypes with a fraction< 1% were excluded from the analysis as they were deemed redundant non-specific T cells in the culture. Assessment of the number of clonotypes detected was performed using the repExplore-function and tracking of clonotypes using the trackClonotypes-function.

### *In silico* calculations on tumor burden and CALRmut specific T cells

We based our calculations on the estimates reported by Sender and Milo (17) on total numbers and turnover of cells in the human organism. The total tumor burden can be computed from the number of neutrophils (N) plus monocytes (M) multiplied by the variant allele frequency (VAF) of mutant CALR multiplied by a factor of 2 since the mutation is heterozygous: 2·(N+M)·VAF.

The number of CALRmut specific T cells is a fraction (b) of the total T cell pool (T), CALRmut specific T cells=b·T. The number of kills per CALR mut specific T cell needed to eradicate the tumor load is then:


2·(N+M)·VAF/b·T.

Each of these kills take a defined period of time, τ. The total time needed for the CALRmut specific T cells to eradicate the tumor load is then:


Total time=2·τ·(N+M)·VAF/b·τ

From Sender and Milo (17) we get the estimates: N=6·10^11^ cells, with a lifetime of 6,6 days and M=1·10^10^ cells with a lifetime of 3,5 days and T=7·10^11^. The neutrophils and monocytes have characteristic lifetimes, hence, the total kill time must be faster than the self-renewal time of tumor cells for successful eradication:


$${\text{τ}}_{\text{total}}\leq 6,6\text{ days}.$$

This relation was plotted for a range of parameter values using Matlab R2020b. The maximal VAF that can be eradicated is then: VAF ≤ 6,6 days·b·T/(2·τ· (N+M))

### Statistics

Statistics were performed using Graphpad Prism version 9 software. ELISPOT responses were evaluated using the distribution free resampling method by Moodie et al. (18) using the statistical software package R.

## Results

### T cells specific for mutant CALR can be enriched for T-cell receptor sequencing using fluorescence activated live cell sorting

**Figure 2:**
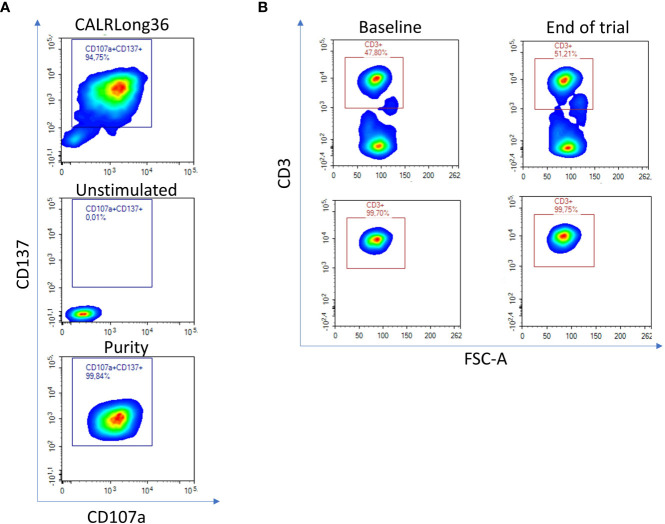
Enrichment of CALRLong36 specific T cells and bone marrow derived T cells using fluorescence activated cell sorting (FACS). **(A)** Four vaccinated CALRmut MPN-patients with a strong immune response to CALRLong36 at the end of trial were chosen for generation of CALRLong36 specific T-cell cultures. Patient peripheral blood mononuclear cells were enriched for CALRmut specific T cells as described in the methods section. The specific T cells were restimulated with peptide and labelled with fluorescence tagged antibodies. Specific cells were enriched as live CD3^+^, CD4^+^, CD107a^+^/CD137^+^ double positive events (patient 1 and 9) or as live CD3^+^, CD8^+^, CD107a^+^/CD137^+^ double positive events (patient 2 and 7) (top). Unstimulated cells were used to set sorting gate (middle). All enriched cells were subjected to purity analysis after enrichment (bottom). **(B)** Cryopreserved bone marrow mononuclear cells from the four patients were thawed and stained with dead cell marker and CD3-APC-H7 for enrichment of T cells derived from bone marrows acquired at baseline and at end-of-trial (top). Purity of the enriched fraction was analyzed post-enrichment (bottom).

Our goal was to clarify if T cells specific to mutant CALR enrich in the bone marrow in patients after completion of therapeutic cancer vaccination against mutant CALR. Hence, four patients that were included in our vaccination trial (11) and displayed strong *in vitro* immune responses to the CALRLong36 vaccination epitope were selected as donors to generate T-cell cultures specific to the CALRLong36 peptide. Patient 1 and 9 (both ET) gave rise to CD4^+^ T-cell cultures, and patient 2 (PMF) and 7 (ET) to CD8^+^ T-cell cultures. The *in vitro* ELISPOT immune responses in these patients can be found in Figure 2A in the report on the vaccine trial (11). Specific T cells from these cultures were enriched using live cell FACS by stimulating specific cells with the CALRLong36 peptide for 18 hours and enriching live T cells co-expressing the T-cell activation markers CD107a and CD137 with unstimulated cells used to set the sorting gate (**Figure 2A**, top and middle). Purity analysis showed satisfactory enrichment (**Figure 2A**, bottom). Next, bone marrow derived T cells were enriched using live cell FACS on cryopreserved bone marrow mononuclear cells acquired at baseline and after trial completion (**Figure 2B**, top) with purity assessed after enrichment (**Figure 2B**, bottom). The quantity and purity of the enriched populations is provided in **Table 1**. The medical records from all patients showed that the acquired bone marrow aspirations were indeed bone marrow aspirations and not peripheral blood, a feat that is not uncommon in patients with MPN. Additionally, the description by the hematopathologists showed that all bone marrow specimens harbored lymphocyte infiltrates.

**Table 1:** Amount and purity of FACS enriched specific T cells and bone marrow derived T cells.

Patient	Amount of specific T cells	Purity of specific T cells (%)	Amount bone marrow derived T cells from baseline sample	Purity of bone marrow derived T cells from baseline sample (%)	Amount bone marrow derived T cells from end-of-trial sample	Purity of bone marrow derived T cells from end-of-trial sample (%)
**1**	2,2 x10^6^	98,9	7,5 x10^5^	99,7	9 x10^5^	99,6
**2**	1,7 x10^5^	99,8	9,5 x10^5^	99,5	1,1 x10^6^	99,5
**7**	1,3 x10^6^	99,8	1,1 x10^6^	99,8	9,6 x10^5^	99,7
**9**	2,2 x10^6^	99,7	6 x10^5^	100	4 x10^5^	99,8

### Patients display a highly diverse T-cell receptor repertoire in the bone marrow

**Figure 3:**
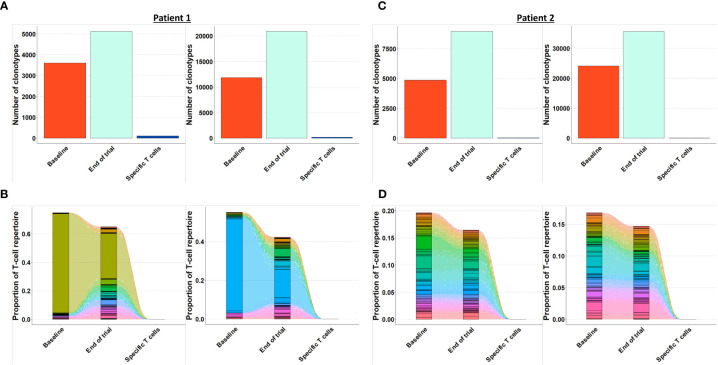
Quantification and clonotype tracking of T cells in patient 1 and 2. **(A)** Quantification of the number of different clonotypes in patient 2 baseline sample, end-of-trial sample and specific T cell sample with α-chain shown to the left and β-chain shown to the right. **(B)** Clonotype tracking of the 100 most prevalent clonotypes in the end-of-trial sample from patient 2 showing tracking trajectories to baseline sample and specific T cells with α-chain shown to the left and β-chain shown to the right. **(C)** Same as A for patient 2. **(D)** Same as B for patient 2.

**Figure 4:**
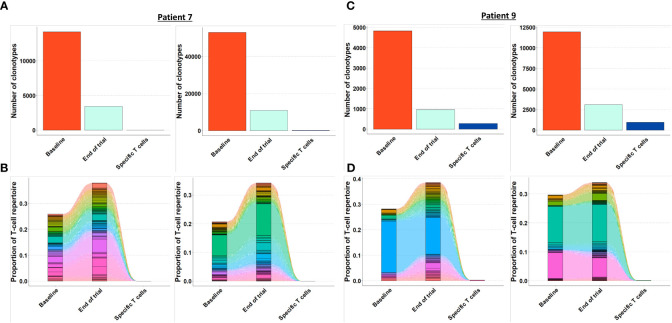
Quantification and clonotype tracking of T cells in patient 7 and 9. **(A)** Quantification of the number of different clonotypes in patient 7 baseline sample, end-of-trial sample and specific T cell sample with α-chain shown to the left and β-chain shown to the right. **(B)** Clonotype tracking of the 100 most prevalent clonotypes in the end-of-trial sample from patient 7 showing tracking trajectories to baseline sample and specific T cells with α-chain shown to the left and β-chain shown to the right. **(C)** Same as A for patient 9. **(D)** Same as B for patient 9.

As the presence of tumor specific T cells in the tumor microenvironment is necessary to attain a clinical response to cancer immune therapy (19), we investigated if T cells specific for mutant CALR enriched in the bone marrow after completion of therapeutic cancer vaccines. Enriched CALRmut specific T cells underwent TCRSeq to identify the CALRmut specific TCR clonotypes. Next, FACS sorted bone marrow derived T cells were analyzed by TCRSeq. Only clonotypes with two or more reads were included in the in *silico* analysis. TCRSeq of bone marrow derived T cells showed a high variation in the frequency of TCRs identified (**Figures 3**, **4** and **Table 2**). For example, only 3601 different α-chains were identified in the sample acquired from patient 1 at baseline (**Figure 3A** and **Table 2**), whereas 52996 different β-chains were identified in the baseline sample from patient 7 (**Figure 4A** and **Table 2**). Naturally, the TCR repertoire from the specific cultures was much lower compared to the bone marrow derived T cells (**Figures 3**, **4**). Low frequency TCRs in the specific T cells could be attributed to background activity from allogenic feeder cells used to expand the specific T cells, and in the downstream analysis it was assumed that only TCRs with a frequency of > 1% in the specific T-cell samples represented CALRmut specific T cells, thereby reducing the amount of TCRs identified in the specific T-cell fraction (**Table 2**).

**Table 2:** Amount of clonotypes in baseline, end-of-trial and specific T cell cultures.

Patient	Chain	N, clonotypes, baseline	N, clonotypes, end of trial	N, clonotypes in specific fraction cells	N, clonotypes in specific fraction > 1% frequency	Fraction of specific clonotypes in baseline sample	Fraction of specific clonotypes in end-of-trial sample	Fraction of specific clonotypes in the specific T cell sample
**1**	α	3601	5113	110	23	0%	0,022%	89,011%
	β	11837	20857	198	22	0%	0,039%	86,089%
**2**	α	4868	8935	25	5	0%	0%	99,028%
	β	24124	35587	27	5	0%	0,001%	99,492%
**7**	α	14159	3420	3	2	0%	0,018%	99,877%
	β	52996	10896	206	1	0%	0%	99,477%
**9**	α	4831	962	279	19	0%	0%	82,825%
	β	11941	3113	958	13	0,016%	0,046%	69,027%

### The T cell repertoire in bone marrow T cells remains constant, and T cells specific to mutant CALR do not enrich in the bone marrow after therapeutic cancer vaccines

Generally, the TCRs in the baseline and end-of-trial samples remained relatively constant as clonotype tracking of the 100 most frequent clonotypes in the end-of-trial samples were present at high frequencies in the baseline samples too (**Figures 3B, D**, **4B, D**).

**Figure 5:**
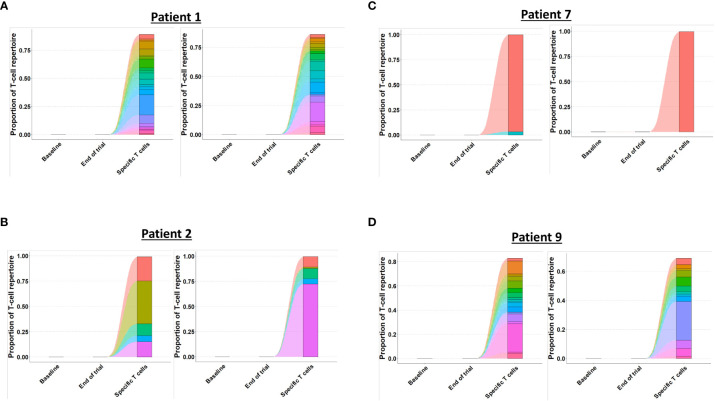
Tracking of clonotypes in CALRmut specific T cells to baseline and end-of-trial samples. **(A)** Patient 1 clonotype tracking with α-chain to the left and β-chain to the right. **(B)** Patient 2 clonotype tracking with α-chain to the left and β-chain to the right. **(C)** Patient 7 clonotype tracking with α-chain to the left and β-chain to the right. **(D)** Patient 9 clonotype tracking with α-chain to the left and β-chain to the right.

Next, we identified TCRs that were present in the specific T-cell cultures with a frequency of > 1% and tracked these clonotypes to the baseline and end-of-trial samples (**Figure 5**). Surprisingly, clonotypes identified in the CALRmut specific T-cell fraction were identified in very low fractions in both baseline and end-of-trial samples (**Figure 5** and **Table 2**). For patient 1, 23 α- and 22 β-chains were identified in the specific T cells. None of these were identified in the baseline sample, but three α− and six β-chains were identified in the end-of-trial bone marrow T cells albeit at a very low frequency (**Table 2** and **Supplementary Material 4**). For patient 2, five α- and five β−chains were identified in the specific T cells, however none of the α−chains were identified in neither the baseline nor the end-of-trial sample. No β-chains from the specific cells were identified in the baseline sample, and only one β-chain was found in the end-of-trial sample at a frequency of 0,001% of all clonotypes (**Table 2** and **Supplementary Material 4**). The specific T- cell culture from patient 7 was almost monoclonal displaying only one β-chain and two α-chains. The β-chain was not identified in neither of the bone marrow samples, and only one of the α-chains was found in the end-of-trial sample at a low frequency of 0,018% (**Table 2** and **Supplementary Material 4**). In the patient 9 derived specific T cells, we managed to detect 19 α-chains and 13 β-chains respectively. Of the former none were detected in neither baseline nor in the end-of-trial samples. Of the latter, one β-chain was identified in the baseline sample at a frequency of 0,016% but was not identified in the end-of-trial sample. Another β-chain from the specific T-cell culture was identified in the end-of-trial sample at a frequency of 0,046%. However, in the specific T cell culture, the clonotype was only the 8^th^ most frequent clonotype and displayed a frequency of only 3,5% of the total T cell repertoire making it unlikely that this clonotype represented a TCR specific to mutant CALR (**Table 1** and **Supplementary Material 4**). Thus overall, we have found that CALRmut specific T cells do not enrich in the bone marrow in patients that have received therapeutic cancer vaccines against mutant CALR.

### CALRLong36 specific T cells recognize autologous *CALR*mut cells from peripheral blood

**Figure 6:**
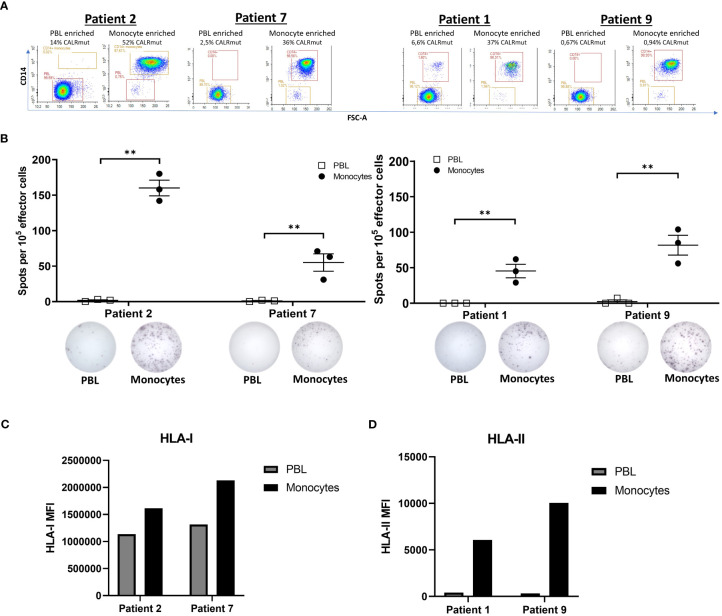
Responses by CALRmut specific T cells to autologous monocytes and peripheral blood lymphocytes. **(A)** Purity analysis of enriched fractions of peripheral blood lymphocytes (PBL) and monocytes with the depiction of the *CALR* mutant variant allele frequency in each enrichment determined by digital droplet PCR. **(B)** Responses by 10^5^ specific T cells stimulated with either 10^4^ autologous PBL or 10^4^ autologous monocytes in an overnight IFN-γ ELISPOT assay. Normalized spot count shown for each patient (top) with representative ELISPOT wells for PBL and monocyte stimulated T cells below. **(C)** Expression of HLA-I by patient 2 and 7 monocytes and PBL was assessed by fluorescent activated cell sorting (FACS). **(D)** Expression of HLA-II by patient 1 and 9 monocytes and PBL was assessed by FACS. Mean fluorescence intensities (MFIs) were calculated by subtracting the MFI of the isotype control from the real staining MFI. Error bars depict standard error of the mean. ** denotes p<0.05 according to the conservative distribution free resampling method (DFR2x) (18).

We have previously demonstrated that CALRmut specific T cells recognize autologous PB myeloid cells and hematopoietic stem cells (HSC) (7, 8). However, the failure to identify enrichment of specific T cells in the bone marrow and target the transformed cells herein could be explained by the fact that the enriched T cells in this study are unable to recognize autologous *CALR*mut cells. Thus, we investigated the recognition of autologous myeloid cells and PBL. These were enriched by FACS (**Figure 6A**) and used as target cells in an IFN-γ ELISPOT assay. In accordance with earlier findings (7, 8), the specific T cells recognized myeloid derived target cells, whereas PBL were not recognized (**Figure 6B**). Analysis by ddPCR confirmed that the monocytes were indeed *CALR*mut with a *CALR*mut VAF ranging from 0,94% to 52%. Surprisingly, all PBL fractions also showed some degree of mutants with a *CALR*mut VAF ranging from 0,67% to 14%. The enriched cells showed a high purity of all fractions, but we cannot rule out the possibility that some PBL fractions were contaminated with monocytes. However, low/absent responses to PBL could also be explained by the lower expression of both HLA-I (**Figure 6C**) and HLA-II (**Figure 6D**) on PBL compared to monocytes.

### The peripheral blood *CALR*mut variant allele frequency is a strong indicator of the number of transformed monocytes and neutrophils suggesting a high tumor burden in patients

Previous data suggests that the *CALR*mut VAF in PB provides a good estimate of PB myeloid cell *CALR*mut VAF (20). Thus, a 40% *CALR*mut VAF in PB indicates a PB myeloid cell *CALR*mut VAF of approximately the same value. As essentially all patients with *CALR*mut are heterozygous (21), this translates into 80% of all myeloid cells carrying the mutation and consequently posing as targets of specific T cells. Both monocytes and neutrophils are produced in high quantities in man (17) implying a high tumor burden in *CALR*mut MPN. Thus, failure of T-cell enrichment in the bone marrow could be explained by an overload of transformed cells in the periphery.

### *In silico* analyses show that cellular immunity is massively outnumbered by peripheral blood *CALR*mut cells

**Figure 7:**
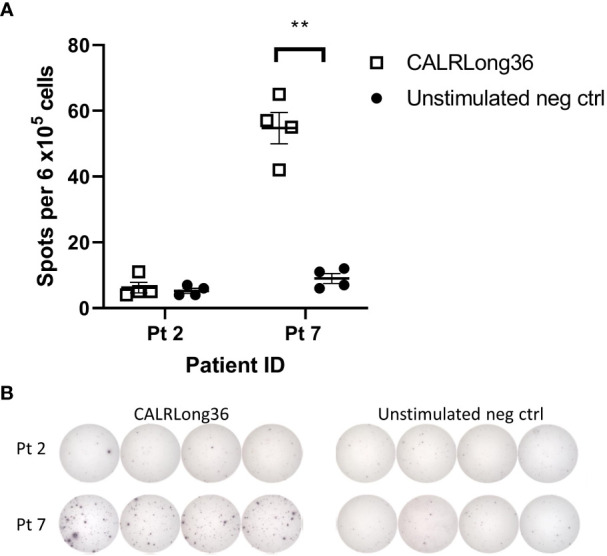
*Ex vivo* IFN-γ ELISPOT responses in patient 2 and 7 against CALRLong36. **(A)** Spot formation in patient 2 and 7 PBMC upon stimulation with the CALRLong36 peptide with unstimulated cells as negative controls. **(B)** Images of *ex vivo* responses in patient 2 and 7 PBMC. Error bars depict standard error of the mean. ** denotes p<0.05 according to the conservative distribution free resampling method (DFR2x) (18).

**Figure 8:**
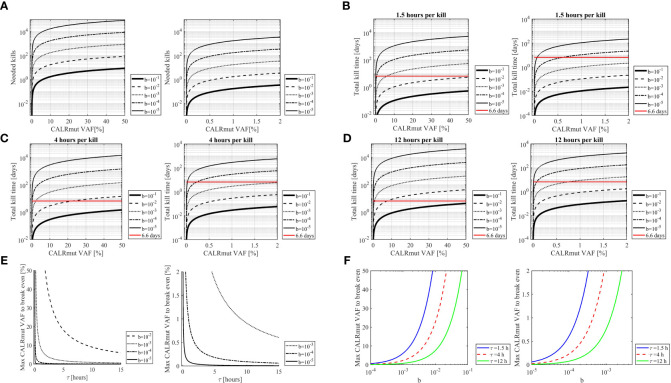
*In silico* analysis of the burden of transformed cells compared to the amount of *CALR*mut specific T cells. **(A)** The amount of needed kills required by each CALRmut specific T cell to curtail the *CALR*mut monocytes and neutrophils as a function of the *CALR*mu*t* variant allele frequency (VAF). The higher the *CALR*mut VAF, the more kills are required by each T cell. Left panel shows *CALR*mut VAF interval of 0-50% with the right panel displaying 0-2% interval for at better resolution of the kills required for a low *CALR*mut VAF. The value b denotes the frequency of CALRmut specific T cells of the entire T cell repertoire and shows that the lower the values of b, the higher number of kills are required per specific T cell. **(B)** The time required for CALRmut specific T cells to kill the *CALR*mut monocytes and neutrophils as a function of the *CALR*mut VAF provided a T cell needs 1,5 hours to kill one transformed cell. The value b denotes the frequency of CALRmut specific T cells of the entire T-cell repertoire. The horizontal line (red) denotes the lifetime of neutrophils i.e., the dominant self-renewal time of transformed cells. Hence, the total kill time must be below this line to allow for eradication of all transformed cells. **(C)** Same as B but with a killing time of 4 hours. **(D)** Same as B and C but with a killing time of 12 hours. **(E)** The maximum allowed *CALR*mut VAF allowed for the cellular immune system to break even with the amount of *CALR*mut monocytes and neutrophils as a function of the time required for each specific T cell to kill a transformed cell. The value b denotes the frequency of CALRmut specific T cells of the entire T-cell repertoire. Left panel shows a *CALR*mut VAF interval of 0-50% with the right panel displaying 0-2% interval for a better resolution of low *CALR*mut VAF. **(F)** The maximum allowed *CALR*mut VAF for the cellular immune system to break even with the amount of *CALR*mut monocytes and neutrophils as a function of the fraction of CALRmut specific T cells in the entire T-cell repertoire. The colored lines depict different values of the time (τ) required for each T cell to kill one transformed cell. Left panel shows a *CALR*mut VAF interval of 0-50% with the right panel displaying 0-2% interval for a better resolution of low *CALR*mut VAF.

Based on assumptions described in the materials section we performed *in silico* analyses to quantify the transformed neutrophils and monocytes in the human organism and compare this to the amount of CALRmut specific T cells. The calculations rely on the fraction of CALRmut specific T cells of the entire T-cell pool (b). An *ex vivo* ELISPOT on PBMC from patient 2 and 7 showed no response in the former, whereas a response was identified in the latter with approximately 45 of 6 x10^5^ assayed cells recognizing the CALRLong36 peptide (**Figure 7**). This provided a fraction of CALRmut specific T cells in the entire T-cell pool (b) of approximately 10^-4^. As patient 2 did not show any *ex vivo* response 10^-5^ was included as a value, as were more optimistic T-cell fractions of 10^-1^, 10^-2^ and 10^-3^. The period of time (τ) a T cell requires to kill a cancer cells was derived from Halle et al. (22) and three different killing times (90 minutes, 4 hours and 12 hours) were assumed. The fraction between the *CALR*mut cells in the periphery and CALRmut specific T cells was plotted displaying the number of kills required by each specific T cell to break even with the number of transformed cells (**Figure 8A**). This showed that even with a high value of b (10^-1^), the amount of *CALR*mut cells outnumbers the T cells. The median *CALR*mut VAF in the vaccination trial was 41%, and calculations showed that even if a highly unrealistic 1/10 of all T cells were specific to mutant CALR, the CALRmut specific T cells would require to kill several *CALR*mut cells. Provided a more realistic fraction of specific T cells of 1/10 000 (b=10^−4^) and a *CALR*mut VAF of 40%, each specific T cell needs to kill approximately 7 000 target cells (**Figure 8A**, left). Another factor to account for is the time required for the T cell to kill the target cell. Thus, we calculated the total kill time required provided different values of τ (**Figures 8B–D**). Assuming a killing time of 4 hours per kill, 10^−4^ specific T cells and a *CALR*mut PB VAF of 40%, approximately 400 days is needed for the cellular immune system to clear the mutant cells (**Figure 8C**, left). Providing a killing time of only 90 minutes, cellular immunity can only match a *CALR*mut VAF< 1%. With τ=90 minutes and b=10^−4^, the maximal VAF that can be eradicated is 0,6% (**Figure 7B**, right). Next, the maximal time a T cell should use to kill a transformed cell to balance the amount of newly formed transformed cells was analyzed (**Figure 8E**). A realistic T-cell fraction of 10^−4^ shows that even with a killing time of only one hour, the maximally allowed VAF that the T cells can control is 1% (**Figure 8E**). Finally, the maximum *CALR*mut VAF which the immune system can control was calculated (**Figure 8F**). Even provided a short killing time and a high fraction of specific T cells, the *CALR*mut VAF is not controllable by T-cell immunity. In conclusion, by using *in silico* analyses we provide data which imply that the levels of transformed cells in *CALR*mut MPN massively exceeds the number of cells which the cellular immune system is able to curtail.

## Discussion

Upon the discovery of the *CALR* exon 9 mutations in MPN it was speculated that the mutations could be targeted by the immune system and thus used for cancer immune therapy (4). Though it was shown that the *CALR* mutations induce a slightly lower expression of HLA-I in cells heterozygous for the mutation (23) which could result in immune escape, *in vitro* experiments demonstrated that autologous *CALR*mut peripheral blood myeloid cells and HSC are targets of CALRmut specific T cells (7, 8). This discovery spurred a therapeutic cancer vaccine trial against mutant CALR (11). The vaccines induced immune responses in PB T cells to mutant CALR but did not induce a clinical response. Even though immune responses can be difficult to compare between studies, it has been shown earlier that therapeutic peptide vaccines induce immune responses to the vaccination antigen which are comparable to the immune response identified in our vaccination trial, and that these immune responses were correlated to a clinical response (24, 25). Dendritic cells from patients with *CALR*mut PMF have been shown to express lower levels of costimulatory molecules which could have a negative impact on the immune response (26). The failure of the immune system to clear the mutant cells in MPN could be attributed to several additional immunosuppressive mechanisms that were just recently reviewed (12). In this study we aimed at identifying whether T cells specific to mutant CALR enrich in the bone marrow after therapeutic cancer vaccinations, as homing of T cells to the tumor microenvironment is important to attain a response to cancer immune therapy (19, 27). TCRSeq on specific T cells and bone marrow T cells showed that specific T cells do not enrich in the bone marrow in vaccinated patients. In addition, we found that the MPN bone marrow is not a T-cell excluded zone and demonstrated a relatively stable TCR repertoire in the bone marrow of patients over time. These data are in good line with a previous study in patients with PMF treated with anti-PD1 antibody pembrolizumab in which the TCR repertoire of PB T cells remained relatively stable (28).

In the current study we confirmed that the specific T cells generated from vaccinated patients recognize *ex vivo* isolated autologous *CALR*mut myeloid cells. ddPCR analysis of both monocytes and PBL showed that the monocytes were *CALR*mut, in addition to a fraction of PBL. This could be explained by either impurities in the enriched PBL, freely circulating DNA from mutant cells lysed during the enrichment procedure, or a fraction of PBL could in fact be *CALR*mut as earlier demonstrated (20). Analysis of HLA-I and HLA-II expression demonstrated a higher expression of both HLA-I and HLA-II by monocytes compared to PBL, which likely explains the higher response to these cells. The fact that CALRmut specific T cells recognize PB monocytes made us hypothesize that even in vaccinated patients, an imbalance between transformed cells and effector T cells in *CALR*mut MPN exists. This was confirmed by *in silico* analyses that were based with assumptions from the literature (17, 22). The fraction of CALRmut specific T cells in the T-cell pool was derived from an *ex vivo* ELISPOT on patient PBMC providing a rough estimate on the amount of T cells specific to mutant CALR of 1/10 000. One should bear in mind however, that the transformed cells may harbor other immunogenic antigens towards which a T-cell response might be evoked after the activation of CALRmut specific T cells. This phenomenon, termed epitope spreading (29), could result in a higher fraction of T cells specific to the transformed cells. Consequently, we included values of the fraction of CALRmut specific T cells in our calculations that might seem unrealistically high. Our results showed that even provided a high fraction of CALRmut specific T cells, each specific T cell would require to kill several hundreds of transformed cells to control the disease. As each T cell needs a certain time to kill a cancer cell, the time factor for elimination of the total pool of transformed cells was calculated. Results showed that in a *CALR*mut patient, T cells require hundreds or even thousands of days to control the transformed cells. Additionally, providing realistic parameters for tumor cell killing time and fraction of specific T cells, the maximum controllable *CALR*mut VAF was shown to be lower than 1%. Importantly, our calculations are based solely on PB myeloid cells and did not take bone marrow HSC into account. Considering the high amount of myeloid cell progenitors in the bone marrow and the fact that the *CALR* exon 9 mutations are identified in early hematopoietic progenitors (3) we envisage that the total burden of *CALR*mut cells is considerably higher than provided in our calculations. Thus, our calculations likely understate the total mass of transformed cells. Based on our findings we believe that the excess of transformed cells in the periphery results in a tumor cell overload for the specific T cells, which could be yet another factor explaining the lack of enrichment of specific T cells into the bone marrow.

Taken into a broader context we cannot completely exclude the possibility that we failed to detect CALRmut specific T cells during the TCRSeq. Firstly, we only included four patients in our analyses. Secondly, our methods to detect specific T cells have some limitations as we enriched and expanded the CALRmut specific T cells by repeated antigen and cytokine stimulations. Thus, our enrichment step could have resulted in a loss of specific cells during the enrichment procedure or during the expansion step. *Ex vivo* isolation of specific T cells by tetramers would have ruled out this factor, however we did not have the necessary tetramers at our disposal. Additionally, one should bear in mind that enrichment by tetramers is not perfect for isolation of specific T cells and tetramers may fail to capture functional T cells (30). The final enrichment was performed on T cells that had been stimulated with CALRLong36 overnight. This could activate non-specific T cells *in vitro* through paracrine stimulation with pro-inflammatory cytokines and these non-specific T cells would be sorted as specific cells. Consequently, TCRs with a frequency< 1% in the bulk cultures were excluded. None of the CALRmut specific TCRs were identified among the most frequent TCRs in the end-of-trial bone marrow specimens, and neither did the specific cells increase from baseline to end of trial. Finally, it cannot be ruled out, that the failure of T-cell homing is explained by the lack of homing receptors on T cells or homing receptor ligands in the bone marrow endothelium – a question that is important to investigate, but outside the scope of the current project. Though there are many other possible explanations beyond the lack of clinical response to the vaccines, we believe that the *in silico* results provided above are noteworthy as they clearly demonstrate a state of tumor cell overload in MPN. Several publications support this notion. Firstly, a phase I trial of the PD1-inhibitor pembrolizumab in 10 patients with PMF, of whom two were *CALR*mut, failed to induce a clinical response (28). This could be ascribed to the lack of immunogenic neo-antigens in the *CALR*wt patients, however Schischlik and coworkers demonstrated that several somatic mutations in MPN generate neo-epitopes with a high HLA-I binding affinity (31). Thus, mutant CALR is potentially not the only target for cellular immunity, yet patients treated with check-point-inhibitors did not show any clinical response to therapy. This lack of response could be explained by the highly immunosuppressive TME in MPN (12, 32), but also by the fact, that the mutant VAF in the reported trial was relatively high reflecting a high peripheral tumor cell load. Of note, T cells from *CALR*mut patients included in the trial recognized mutant CALR underscoring that CALRmut specific T cells were present in these patients but failed to induce a clinical response (33). Secondly, a murine vaccination study in a del52 knock-in model of mutant CALR failed to induce a reduction in the *CALR*mut VAF. The lack of effect was ascribed to poor T-cell responses to the vaccination epitopes, however the high tumor load could also explain the lack of effect on the *CALR*mut VAF (34). Thirdly, another *in vivo* murine vaccination study in a *CALR*mut model demonstrated a clinical response to the vaccines. This study employed a different treatment schedule than the former, but the model also differed as the tumor cells were *CALR*mut transduced lymphoma cells (8). The important difference here being that the lymphoma model is a compartmentalized model of *CALR*mut MPN, whereas the del52 knock-in is a global model of MPN. In the former model, the *CALR*mut cells are present in a limited number, confined to the tumor and thereby present in a limited number whereas in the latter, transformed cells are present in the entire organism and at a much higher level.

The findings in the present study fits well with data on cancer immune therapy in other hematological cancers. In relapsed/refractory B cell acute lymphoblastic leukemia, an increased tumor burden is negatively associated with survival in patients treated with chimeric antigen receptor T cells (35). In acute myeloid leukemia (AML) and multiple myeloma an increased tumor burden is negatively correlated to overall survival in patients treated with allogeneic hematopoietic stem cell transplantation (allo-HSCT) (36, 37). To our knowledge the impact of the tumor burden on response to allo-HSCT in PMF has not been investigated. Hence, we are currently investigating this in a retrospective study in patients with PMF treated with allo-HSCT.

In conclusion we have shown that therapeutic cancer vaccination against mutant CALR does not result in enrichment of mutation specific T cells in the bone marrow. The recognition and killing of bone marrow resident *CALR*mut HSC is of utmost importance to attain a response to cancer immune therapy in MPN as killing of the transformed HSC in the bone marrow will prevent entry of more mature subsets into the peripheral blood and ultimately cure the patient. The lack of effect to therapeutic cancer vaccination against mutant CALR could be explained by the highly immunosuppressive TME in MPN. However, based on our *in vitro* and in *silico* experiments we speculate that the high tumor burden in PB of patients with MPN outcompetes cellular immunity. Thus, we envisage high tumor load and high tumor cell turnover as an additional immunosuppressive mechanism in MPN that needs to be accounted upon designing cancer immune therapy for patients with MPN.

## Data availability statement

The datasets presented in this article are not readily available because of the Danish Law on data protection and the GDPR rules. Requests to access the datasets should be directed to the corresponding author.

## Ethics statement

The studies involving human participants were reviewed and approved by Regional Ethics Committee for Zealand Region, Denmark Approval number SJ-680. The patients/participants provided their written informed consent to participate in this study.

## Author contributions

MH conceived the project, performed experiments, analyzed data, and wrote the manuscript. MA performed experiments, analyzed data, and wrote the manuscript. ST performed experiments, analyzed data, and wrote the manuscript. SA performed experiments. TL performed experiments and wrote the manuscript. JH performed experiments. VS and LK performed experiments and analyzed data. JO performed experiments and analyzed the data. MG analyzed the data and provided vital reagents. HH conceived the project and analyzed data. MHA conceived the project, analyzed data, and wrote the manuscript. All authors contributed to the article and approved the submitted version.

## References

[1] Campbell PJ Green AR. The myeloproliferative disorders. N Engl J Med (2006) 355:2452–66. doi: 10.1056/NEJMra063728 17151367

[2] Kralovics R Passamonti F Buser AS Teo S-S Tiedt R Passweg JR. A gain-of-function mutation of JAK2 in myeloproliferative disorders. N Engl J Med (2005) 352:1779–90. doi: 10.1056/NEJMoa051113 15858187

[3] Nangalia J Massie CE Baxter EJ Nice FL Gundem G Wedge DC. Somatic CALR mutations in myeloproliferative neoplasms with nonmutated JAK2. N Engl J Med (2013) 369:2391–405. doi: 10.1056/NEJMoa1312542 PMC3966280 24325359

[4] Klampfl T Gisslinger H Harutyunyan AS Nivarthi H Rumi E Milosevic JD. Somatic mutations of calreticulin in myeloproliferative neoplasms. N Engl J Med (2013) 369:2379–90. doi: 10.1056/NEJMoa1311347 24325356

[5] Pietra D Rumi E Ferretti VV Buduo CAD Milanesi C Cavalloni C. Differential clinical effects of different mutation subtypes in CALR-mutant myeloproliferative neoplasms. Leukemia (2016) 30:431–8. doi: 10.1038/leu.2015.277 PMC4740452 26449662

[6] Holmström MO Riley CH Svane IM Hasselbalch HC Andersen MH. The CALR exon 9 mutations are shared neoantigens in patients with CALR mutant chronic myeloproliferative neoplasms. Leukemia (2016) 30:2413–6. doi: 10.1038/leu.2016.233 27560107

[7] Holmström MO Martinenaite E Ahmad SM Met Ö Friese C Kjær L. The calreticulin (CALR) exon 9 mutations are promising targets for cancer immune therapy. Leukemia (2018) 32:429–37. doi: 10.1038/leu.2017.214 28676668

[8] Gigoux M Holmström MO Zappasodi R Park JJ Pourpe S Bozkus CC. Calreticulin mutant myeloproliferative neoplasms induce MHC-I skewing, which can be overcome by an optimized peptide cancer vaccine. Sci Transl Med (2022) 14. doi: 10.1126/scitranslmed.aba4380 PMC11182673 35704596

[9] Holmström MO Ahmad SM Klausen U Bendtsen SK Martinenaite E Riley CH. High frequencies of circulating memory T cells specific for calreticulin exon 9 mutations in healthy individuals. Blood Cancer J (2019) 9. doi: 10.1038/s41408-018-0166-4 PMC6336769 30655510

[10] Holmström MO Cordua S Skov V Kjær L Pallisgaard N Ellervik C. Evidence of immune elimination, immuno-editing and immune escape in patients with hematological cancer. Cancer Immunol Immunother (2020) 69:315–24. doi: 10.1007/s00262-019-02473-y PMC11027882 31915854

[11] Handlos Grauslund J Holmström MO Jørgensen NG Klausen U Weis-Banke SE El Fassi D. Therapeutic cancer vaccination with a peptide derived from the calreticulin exon 9 mutations induces strong cellular immune responses in patients with CALR-mutant chronic myeloproliferative neoplasms. Front Oncol (2021) 11:637420. doi: 10.3389/fonc.2021.637420 33718228 PMC7952976

[12] Holmström MO Hasselbalch HC Andersen MH. Cancer immune therapy for Philadelphia chromosome-negative chronic myeloproliferative neoplasms. Cancers (Basel) (2020) 12:1–21. doi: 10.3390/cancers12071763 PMC7407874 32630667

[13] Holmström MO Andersen MH. Healthy donors harbor memory T cell responses to RAS neo-antigens. Cancers (Basel) (2020) 12:1–16. doi: 10.3390/cancers12103045 PMC7589254 33086698

[14] Kjær L Cordua S Holmström MO Thomassen M Kruse TA Pallisgaard N. Differential dynamics of CALR mutant allele burden in myeloproliferative neoplasms during interferon alfa treatment. PloS One (2016) 11:e0165336. doi: 10.1371/journal.pone.0165336 27764253 PMC5072743

[15] Cordua S Kjaer L Skov V Pallisgaard N Hasselbalch HC Ellervik C. Prevalence and phenotypes of JAK2 V617F and Calreticulin mutations in a Danish general population. Blood (2019) 134:469–79. doi: 10.1182/blood.2019001113 31217187

[16] ImmunoMind Team. immunarch: an R Package for Painless Bioinformatics Analysis of T-Cell and B-Cell Immune Repertoires. (2019). doi: 10.5281/zenodo.3367200

[17] Sender R Milo R. The distribution of cellular turnover in the human body. Nat Med (2021) 27:45–8. doi: 10.1038/s41591-020-01182-9 33432173

[18] Moodie Z Price L Gouttefangeas C Mander A Janetzki S Löwer M. Response definition criteria for ELISPOT assays revisited. Cancer Immunol Immunother (2010) 59:1489–501. doi: 10.1007/s00262-010-0875-4 PMC2909425 20549207

[19] Sackstein R Schatton T Barthel SR. T-lymphocyte homing: An underappreciated yet critical hurdle for successful cancer immunotherapy. Lab Investig (2017) 97:669–97. doi: 10.1038/labinvest.2017.25 PMC5446300 28346400

[20] Kjaer L Orebo Holmström M Cordua S Andersen MH Svane IM Thomassen M. Sorted peripheral blood cells identify CALR mutations in B- and T-lymphocytes. Leuk Lymphoma (2017) 59:973–7. doi: 10.1080/10428194.2017.1359743 28792253

[21] Jeromin S Kohlmann A Meggendorfer M Schindela S Perglerova K Nadarajah N. Next-generation deep-sequencing detects multiple clones of CALR mutations in patients with BCR-ABL1 negative MPN. Leukemia (2016) 30:973–6. doi: 10.1038/leu.2015.207 26220041

[22] Halle S Halle O Förster R. Mechanisms and dynamics of T cell-mediated cytotoxicity *in vivo* . Trends Immunol (2017) 38:432–43. doi: 10.1016/j.it.2017.04.002 28499492

[23] Arshad N Cresswell P. Tumor-associated calreticulin variants functionally compromise the peptide loading complex and impair its recruitment of MHC-I. J Biol Chem (2018) 293:9555–69. doi: 10.1074/jbc.RA118.002836 PMC6016473 29769311

[24] Iversen TZ Engell-Noerregaard L Ellebaek E Andersen R Larsen SK Bjoern J. Long-lasting disease stabilization in the absence of toxicity in metastatic lung cancer patients vaccinated with an epitope derived from indoleamine 2,3 dioxygenase. Clin Cancer Res (2014) 20:221–32. doi: 10.1158/1078-0432.CCR-13-1560 24218513

[25] Rahma OE Hamilton JM Wojtowicz M Dakheel O Bernstein S Liewehr DJ. The immunological and clinical effects of mutated ras peptide vaccine in combination with IL-2, GM-CSF, or both in patients with solid tumors. J Transl Med (2014) 12:1–12. doi: 10.1186/1479-5876-12-55 24565030 PMC3942063

[26] Romano M Sollazzo D Trabanelli S Barone M Polverelli N Perricone M. Mutations in JAK2 and Calreticulin genes are associated with specific alterations of the immune system in myelofibrosis. Oncoimmunology (2017) 6. doi: 10.1080/2162402X.2017.1345402 PMC5665081 29123956

[27] Franciszkiewicz K Boissonnas A Boutet M Combadière C Mami-Chouaib F. Role of chemokines and chemokine receptors in shaping the effector phase of the antitumor immune response. Cancer Res (2012) 72:6325–32. doi: 10.1158/0008-5472.CAN-12-2027 23222302

[28] Hobbs G Bozkus CC Moshier E Dougherty M Bar-Natan M Sandy L. PD-1 inhibition in advanced myeloproliferative neoplasms. Blood Adv (2021) 5:5086–97. doi: 10.1182/BLOODADVANCES.2021005491 PMC9152999 34581778

[29] Gulley JL Madan RA Pachynski R Mulders P Sheikh NA Trager J. Role of antigen spread and distinctive characteristics of immunotherapy in cancer treatment. J Natl Cancer Inst (2017) 109:1–9. doi: 10.1093/jnci/djw261 PMC5441294 28376158

[30] Dolton G Tungatt K Lloyd A Bianchi V Theaker SM Trimby A. More tricks with tetramers: A practical guide to staining T cells with peptide-MHC multimers. Immunology (2015) 146. doi: 10.1111/imm.12499 PMC4552497 26076649

[31] Schischlik F Jäger R Rosebrock F Hug E Schuster M Holly R. Mutational landscape of the transcriptome offers putative targets for immunotherapy of myeloproliferative neoplasms. Blood (2019) 134:199–210. doi: 10.1182/blood.2019000519 31064751 PMC6624966

[32] Barosi G. An immune dysregulation in MPN. Curr Hematol Malig Rep (2014) 9:331–9. doi: 10.1007/s11899-014-0227-0 25139710

[33] Bozkus CC Roudko V Finnigan JP Mascarenhas J Hoffman R Iancu-Rubin C. Immune checkpoint blockade enhances shared neoantigen-induced T-cell immunity directed against mutated calreticulin in myeloproliferative neoplasms. Cancer Discov (2019) 9:1192–207. doi: 10.1158/2159-8290.CD-18-1356 PMC6726533 31266769

[34] Balligand T Achouri Y Pecquet C Gaudray G Colau D Hug E. Knock-in of murine Calr del52 induces essential thrombocythemia with slow-rising dominance in mice and reveals key role of Calr exon 9 in cardiac development. Leukemia (2020) 34:510–21. doi: 10.1038/s41375-019-0538-1 31471561

[35] Li M Xue SL Tang X Xu J Chen S Han Y. The differential effects of tumor burdens on predicting the net benefits of ssCART-19 cell treatment on r/r B-ALL patients. Sci Rep (2022) 12. doi: 10.1038/s41598-021-04296-3 PMC8748521 35013456

[36] Ogawa H Ikegame K Daimon T Uchida N Fukuda T Kakihana K. Impact of pretransplant leukemic blast% in bone marrow and peripheral blood on transplantation outcomes of patients with acute myeloid leukemia undergoing allogeneic stem cell transplantation in non-CR. Bone Marrow Transplant (2018) 53:478–82. doi: 10.1038/s41409-017-0028-x 29330394

[37] Mosebach J Shah S Delorme S Hielscher T Goldschmidt H Schlemmer HP. Prognostic significance of tumor burden assessed by whole-body magnetic resonance imaging in multiple myeloma patients treated with allogeneic stem cell transplantation. Haematologica (2018) 103:336–43. doi: 10.3324/haematol.2017.176073 PMC5792278 29217779

