# Supplementary material for: Therapeutic cancer vaccination against mutant calreticulin in myeloproliferative neoplasms induces expansion of specific T cells in the periphery but specific T cells fail to enrich in the bone marrow
Source: Front Immunol. 2023 Aug 17;14:1240678. doi: 10.3389/fimmu.2023.1240678 (PMC10470021; doi:10.3389/fimmu.2023.1240678)
Supplement: Supplementary file 1 [file DataSheet_1.pdf]

Supplementary Material

T cells specific to mutant calreticulin expand in the periphery but fail to enrich in the bone marrow after therapeutic cancer vaccination

Morten Orebo Holmström[1,2]*, Morten Andersen[3], Sofie Traynor[4], Shamaila Munir Ahmad[1], Thomas Landkildehus Lisle[1], Jacob Handlos Grauslund[1], Vibe Skov[6], Lasse Kjær[6], Johnny T. Ottesen[3], Morten Frier Gjerstorff[4,5], Hans Carl Hasselbalch[6], Mads Hald Andersen[1,2]

*** Correspondence:** Corresponding Author: morten.orebo.holmstroem@regionh.dk

Supplementary Material 1

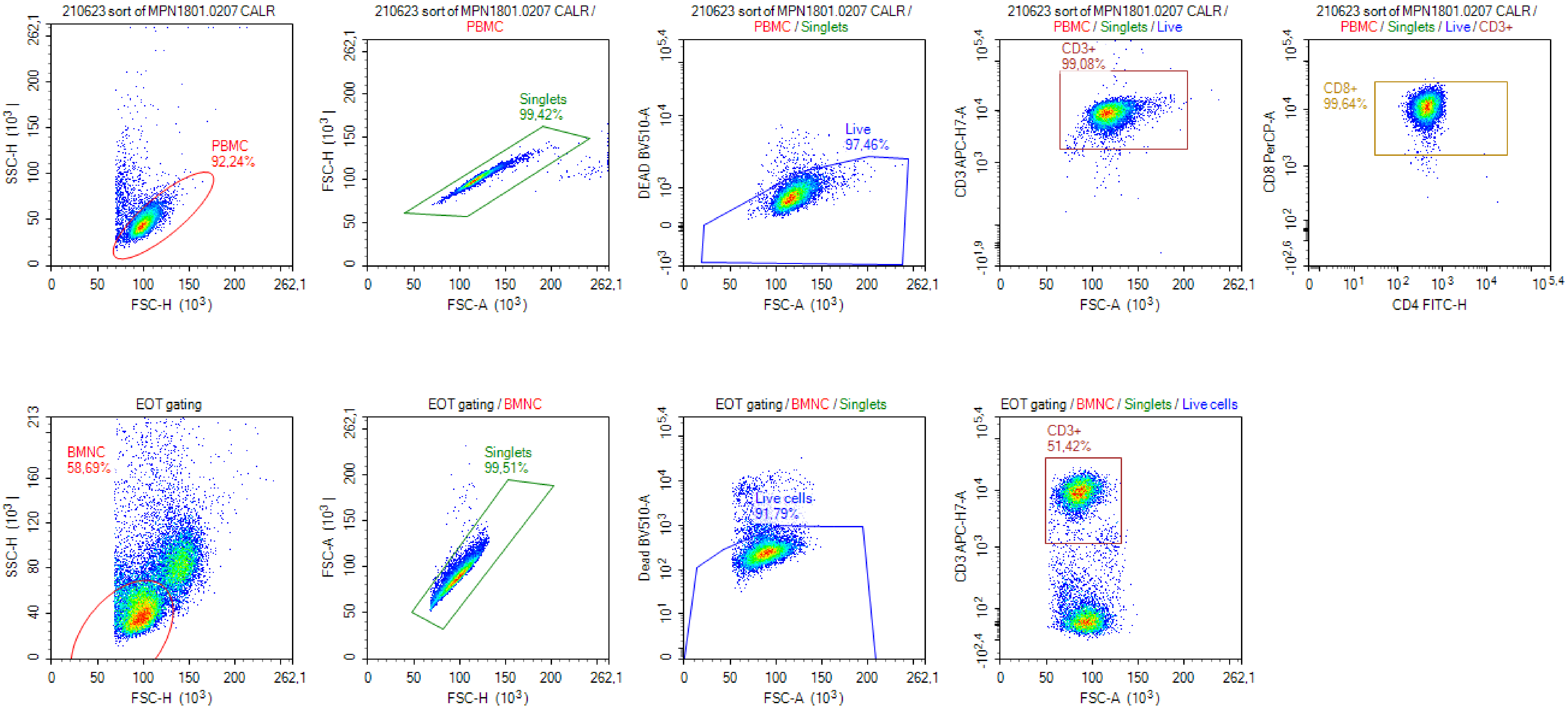

Supplementary Material 2

Experiment	Antigen	Fluorophore	Provider
**Isolation of CALRmut specific T cells**	CD3	APC-H7	BD Biosciences, San José, CA, USA
	CD4	FITC or PerCP	BD Biosciences, San José, CA, USA
	CD8	PerCP or FITC	BD Biosciences, San José, CA, USA
	Dead cell marker (FVS510)	BV510	BD Biosciences, San José, CA, USA
	CD107a	PE	BD Biosciences, San José, CA, USA
	CD137	BV421	BD Biosciences, San José, CA, USA
**Isolation bone marrow derived T cells**			
	Dead cell marker (FVS510)	BV510	BD Biosciences, San José, CA, USA
	CD3	APC-H7	BD Biosciences, San José, CA, USA
**FACS of live monocytes and peripheral blood lymphocytes**			
	Dead cell marker (FVS510)	BV510	BD Biosciences, San José, CA, USA
	CD14	PerCP	BD Biosciences, San José, CA, USA
**Expression of HLA-I and HLA-II by monocytes and lymphocytes**			
	CD14	BV421	Biolegend, San Diego, CA, USA
	CD14	PerCP	BD Biosciences, San José, CA, USA
	HLA-I	PE	BD Biosciences, San José, CA, USA
	HLA-II	FITC	BD Biosciences, San José, CA, USA
	Dead cell marker (FVS510)	BV510	BD Biosciences, San José, CA, USA

Supplementary Material 3

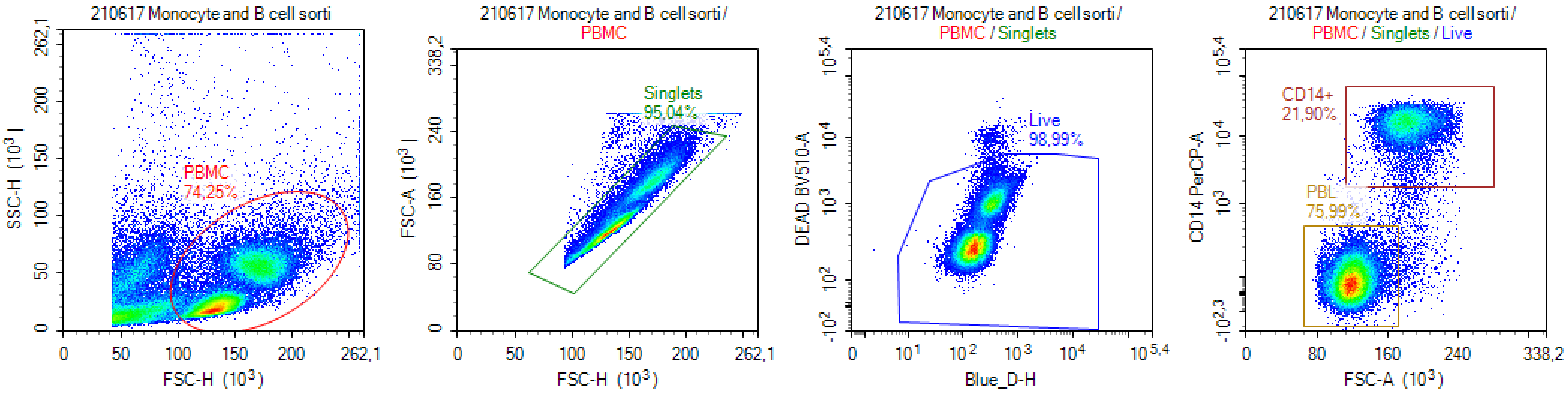

Supplementary Material 4

Patient 1, alpha chain

Amino acid sequence	Baseline, %	End of trial, %	Specific T cells, %
CAVSYYNTDKLIF	0,000	0,000	17,710
CAPGGSNYKLTF	0,000	0,000	7,859
CGTANQAGTALIF	0,000	0,000	7,468
CALEAQAGTALIF	0,000	0,000	6,830
CAVDYGNNRLAF	0,000	0,000	5,642
CALSGEFNDYKLSF	0,000	0,009	5,529
CAVEAGYGGATNKLIF	0,000	0,000	4,866
CAVSRWGDDKIIF	0,000	0,000	4,805
CAEKDDYKLSF	0,000	0,004	4,050
CLVGDQPDNNARLMF	0,000	0,000	2,936
CAVSDWGYQKVTF	0,000	0,000	2,343
CAASSGDKLTF	0,000	0,000	2,305
CAVDENSGNTPLVF	0,000	0,000	2,220
CAVDMDTDKLIF	0,000	0,000	2,201
CAMTSGNTPLVF	0,000	0,000	2,046
CAVETRHTGGFKTIF	0,000	0,000	1,939
CAACYYNTDKLIF	0,000	0,000	1,356
CAALIYNTDKLIF	0,000	0,000	1,310
CAMSSPWGADKLIF	0,000	0,000	1,264
CVVSAVKGASTDKLIF	0,000	0,009	1,119
CAGSDYKLSF	0,000	0,000	1,075
CAENGGNNARLMF	0,000	0,000	1,071
CAATSLDLRGNNRLAF	0,000	0,000	1,066
Total	0,000	0,022	89,011

Patient 1, beta chain

Amino acid sequence	Baseline, %	End of trial, %	Specific T cells, %
CASSYVQGGGETQYF	0,000	0,000	16,569
CASSTYGDTDTQYF	0,000	0,007	8,908
CASSQAVETQYF	0,000	0,000	8,066
CASSSGTSSYPGDETQYF	0,000	0,000	7,093
CASSYSEGGSEKLFF	0,000	0,000	5,437
CATSRDQRYTGELFF	0,000	0,002	5,378
CASSPHGLTIEAFF	0,000	0,000	5,268
CASSGDSSTQYF	0,000	0,006	3,096
CASSSSGIYNEQFF	0,000	0,000	2,639
CASRPGEGRYEQYF	0,000	0,000	2,567
CATSDADRSSYNEQFF	0,000	0,000	2,400
CASSETGLGPDTQYF	0,000	0,000	2,338
CASSLTGQETQYF	0,000	0,004	2,094
CASSLGTSGSRHTGELFF	0,000	0,000	2,013
CASSEVGSGSGDTQYF	0,000	0,000	1,999
CSALGGANIQYF	0,000	0,000	1,931
CATSPREGAFGYTF	0,000	0,016	1,582
CASSWGAGGATDTQYF	0,000	0,005	1,575
CASSPMVDNEQFF	0,000	0,000	1,441
CASSWVQGYGYTF	0,000	0,000	1,385
CASSLLGGNEKLFF	0,000	0,000	1,205
CASSEGVGGGVDEQFF	0,000	0,000	1,114
Total	0,000	0,039	86,089

Patient 2, alpha chain

Amino acid sequence	Baseline, %	End of trial, %	Specific T cells, %
CAGETSYDKVIF	0	0	23,915
CALKYNNDMRF	0	0	42,355
CALQNFGNEKLTF	0	0	11,589
CAVLKTSYDKVIF	0	0	6,022
CAYRPWDDKIIF	0	0	15,145
Total	0	0	99,028

Patient 2, beta chain

Amino acid sequence	Baseline, %	End of trial, %	Specific T cells, %
CSARKVLAGNFEQYF	0,000	0,000	72,294
CASSDRGDTDTQYF	0,000	0,000	10,257
CASSPDAGTGNTIYF	0,000	0,000	10,225
CASTLGGAGGGDTQYF	0,000	0,000	5,206
CASSGGLRGSYNEQFF	0,000	0,001	1,511
Total	0,000	0,001	99,492

Patient 7, alpha chain

Amino acid sequence	Baseline, %	End of trial, %	Specific T cells, %
CALYSQGGSEKLVF	0	0,000	96,547
CAYRFWDNARLMF	0	0,018	3,329
Total	0	0,018	99,877

Patient 7, beta chain

Amino acid sequence	Baseline, %	End of trial, %	Specific T cells, %
CASSEALAWETQYF	0	0	99,477
Total	0	0	99,477

Patient 9, alpha chain

Amino acid sequence	Baseline, %	End of trial, %	Specific T cells, %
CAASEEDDMRF	0,000	0,000	23,391
CAETMFNDYKLSF	0,000	0,000	10,431
CVVIDYNTDKLIF	0,000	0,000	6,227
CALSHNMDTGRRALTF	0,000	0,000	6,012
CILRYRILNTGNQFYF	0,000	0,000	4,295
CAATNDNFNKFYF	0,000	0,000	4,251
CASLNTGNQFYF	0,000	0,000	4,052
CAPLKAAGNKLTF	0,000	0,000	3,661
CALSDPENQAGTALIF	0,000	0,000	3,634
CAYRRVEGNEKLTF	0,000	0,000	3,468
CAEILNTNAGKSTF	0,000	0,000	2,292
CAALMYNTDKLIF	0,000	0,000	1,878
CLVGDDQQAAGNKLTF	0,000	0,000	1,623
CAVEAGRMWGAQKLVF	0,000	0,000	1,612
CATDSRDDKIIF	0,000	0,000	1,520
CALGTNAGKSTF	0,000	0,000	1,254
CAVVGNQGGKLIF	0,000	0,000	1,128
CAETSDDYKLSF	0,000	0,000	1,090
CAASGGSNYKLTF	0,000	0,000	1,006
Total	0,000	0,000	82,825

Patient 9, beta chain

Amino acid sequence	Baseline, %	End of trial, %	Specific T cells, %
CASSPSRWSGQYQETQYF	0,000	0,000	26,725
CASSEVGLAADTQYF	0,000	0,000	6,142
CASSYVQGGQETQYF	0,000	0,000	5,597
CASVGGDFGANVLTF	0,000	0,000	5,427
CASSEVGGPYEQYF	0,000	0,000	4,562
CASNSGLSGYTF	0,000	0,000	4,385
CASSLRFYSPLHF	0,000	0,000	3,982
CASSLYSAQIAGETDTQYF	0,000	0,046	3,563
CASRGTSGVYTQYF	0,000	0,000	2,683
CASSLSGGGSTYNEQFF	0,000	0,000	1,651
CATSRDHYGYTF	0,000	0,000	1,519
CASSLVGAGGSDTQYF	0,000	0,000	1,497
CASRRLAGRYNEQFF	0,016	0,000	1,294
Total	0,016	0,046	69,027

Supplementary Material Legends

Supplementary Material 1: Gating strategy for enrichment of CALRLong36 specific T cells (top), and gating strategy for enrichment of bone marrow derived T cells (bottom).

Supplementary Material 2: Antibody panels used for the different FACS experiments.

Supplementary Material 3: Gating strategy for sorting of PBL and monocytes.

Supplementary Material 4. Frequencies of CALRmut specific T-cell receptors (TCR) in baseline, end-of-trial and CALRmut specific T cell cultures for both α- and β-chains for all four patients.
